# CT60 genotype does not affect CTLA-4 isoform expression despite association to T1D and AITD in northern Sweden

**DOI:** 10.1186/1471-2350-8-3

**Published:** 2007-02-06

**Authors:** Sofia Mayans, Kurt Lackovic, Caroline Nyholm, Petter Lindgren, Karin Ruikka, Mats Eliasson, Corrado M Cilio, Dan Holmberg

**Affiliations:** 1 Medical and Clinical Genetics, Dept. of Medical Biosciences, Umeå University SE-90185 Umeå, Sweden; 2 Cellular Autoimmunity Unit, Dept. of Clinical Sciences, Malmö University Hospital, Lund University, SE-20502, Malmö, Sweden; 3 Department of Medicine, Sunderby Hospital, SE-97180 Luleå, Sweden; 4 Department of Public Health and Clinical Medicine, Umeå University SE-90185 Umeå, Sweden

## Abstract

**Background:**

Polymorphisms in and around the *CTLA-4 *gene have previously been associated to T1D and AITD in several populations. One such single nucleotide polymorphism (SNP), CT60, has been reported to affect the expression level ratio of the soluble (sCTLA-4) to full length CTLA-4 (flCTLA-4) isoforms. The aims of our study were to replicate the association previously published by Ueda *et al*. of polymorphisms in the *CTLA-4 *region to T1D and AITD and to determine whether the CT60 polymorphism affects the expression level ratio of sCTLA-4/flCTLA-4 in our population.

**Methods:**

Three SNPs were genotyped in 253 cases (104 AITD cases and 149 T1D cases) and 865 ethnically matched controls. Blood from 23 healthy individuals was used to quantify mRNA expression of CTLA-4 isoforms in CD4^+ ^cells using real-time PCR. Serum from 102 cases and 59 healthy individuals was used to determine the level of sCTLA-4 protein.

**Results:**

Here we show association of the MH30, CT60 and JO31 polymorphisms to T1D and AITD in northern Sweden. We also observed a higher frequency of the CT60 disease susceptible allele in our controls compared to the British, Italian and Dutch populations, which might contribute to the high frequency of T1D in Sweden. In contrast to previously published findings, however, we were unable to find differences in the sCTLA-4/flCTLA-4 expression ratio based on the CT60 genotype in 23 healthy volunteers, also from northern Sweden. Analysis of sCTLA-4 protein levels in serum showed no correlation between sCTLA-4 protein levels and disease status or CT60 genotype.

**Conclusion:**

Association was found between T1D/AITD and all three polymorphisms investigated. However, in contrast to previous investigations, sCTLA-4 RNA and protein expression levels did not differ based on CT60 genotype. Our results do not rule out the CT60 SNP as an important polymorphism in the development of T1D or AITD, but suggest that further investigations are necessary to elucidate the effect of the *CTLA-4 *region on the development of T1D and AITD.

## Background

Autoimmune thyroid disease (AITD) and type 1 diabetes mellitus (T1D) are autoimmune disorders under complex genetic control that develop through a process mediated by T lymphocytes [1]. Association between T1D and AITD is well known, and these diseases frequently cluster within the same family and in the same individual [2] indicating common genetic causes [1,3,4]. Risk of both T1D and AITD has been linked to the cytotoxic T-lymphocyte associated antigen-4 (*CTLA-4*) region on chromosome 2q33 in many populations including the Swedish, Italian and Finnish [5-7]. Fine mapping in and around the *CTLA-4 *gene performed by Ueda *et al*. [8] narrowed down the region of association to single nucleotide polymorphisms (SNPs) 3' of the *CTLA-4 *and 5' of the inducible co-stimulator (*ICOS*) genes. The CT60 polymorphism (rs30807243), located 3' to the known polyadenylation site of *CTLA-4*, showed the highest association to autoimmune disease. Investigation of full-length CTLA-4 (flCTLA-4) and soluble CTLA-4 (sCTLA-4) [8,9] expression based on the CT60 genotype revealed a lower expression of sCTLA-4 in individuals homozygous for the G allele in the UK population [8]. In line with this notion we previously analyzed a large pedigree from northern Sweden in which multiple individuals suffered T1D and AITD. Applying a model in which all T1D and AITD patients were considered affected, evidence in form of linkage to the MHC and the *CTLA-4 *loci was obtained [5]. Based on this background, we set out to investigate the previously published[8] association of three polymorphisms in the *CTLA-4 *region to T1D and AITD in the population of northern Sweden and subsequently investigate the expression of flCTLA-4, sCTLA-4 and ICOS based on the CT60 genotype.

## Methods

### Subjects

Three SNPs adjacent to the *CTLA-4 *gene, MH30 (rs231806), CT60 (rs30807243) and JO31 (rs11571302) were genotyped in 1118 subjects from northern Sweden (104 patients with AITD, 149 patients with T1D and 865 matched controls). Cases were identified through an autoimmune disease register at Sunderby hospital in northern Sweden. Healthy volunteers from northern Sweden were used as ethnically matched controls. The study was carried out with the approval of the regional ethical review board and informed consent was obtained from all participants.

Blood from 23 healthy individuals (relatives to cases in the Sunderby autoimmune disease register) was used to measure the expression of flCTLA-4, sCTLA-4 and ICOS.

Serum from 59 healthy individuals, 43 individuals with AITD, 46 individuals with T1D and 13 individuals with both AITD and T1D was used to determine the level of sCTLA-4 protein.

### DNA extraction

Genomic DNA from cases was extracted from mouth swabs. Detailed instructions for mouth swab sampling were sent to each individual with a kit containing all necessary reagents. Mouth swabbing was performed by rubbing the inside of the cheek with a small brush for approximately 30 sec. The brush was then placed in 600 μl 50 mM NaOH and returned to the laboratory by standard mail. Mouth swabs were vortexed shortly before incubation at 95°C for 10 min. After incubation the mouth swabs were vortexed again, the brush was removed from each tube, 50 μl 1 M Tris-HCl (pH 8.0) was added to each tube and the tubes were stored at 4°C.

Genomic DNA from controls was extracted from whole blood using standard phenol-chisam methods.

### Genotyping of polymorphisms in close proximity to *CTLA-4*

The three SNPs of interest were genotyped using TaqMan Assay-by-Design (Applied Biosystems, Foster City, CA, USA). Assays were performed according to the manufacturer's instructions. MH30 is located 5' of the CTLA-4 transcript and both CT60 and JO31 are located 3' of the CTLA-4 transcript. Genotypes were analyzed using the allelic discrimination function of the TaqMan 7900 HT Fast Real-Time PCR system (Applied Biosystems, Foster City, CA, USA). 8% of the total samples were run in duplicate for each SNP, and 100% concordance was observed between duplicates. A genotyping success rate from 89.9% to 99.5% (Table 1) was obtained for the SNPs under investigation.

**Table 1:** Significant association of MH30, CT60 and JO31 to autoimmune disease in northern Sweden.

**MH30**	**Case (%)**	**Control (%)**	**OR (95%CI)**	**P value**
CC	16 (6.7)	108 (13.9)		
CG	111 (46.4)	362 (46.6)		
GG	112 (46.9)	307 (39.5)		
CG vs. CC			2.07 (1.18–3.65)	0.01
GG vs. CC			2.46 (1.40–4.35)	0.002
Multiplicative model			1.40 (1.12–1.75)	0.004
Success rate (%)	94.5	89.9		
				
**CT60**				
AA	15 (6.4)	117 (14.4)		
AG	111 (47.2)	394 (48.5)		
GG	109 (46.4)	301 (37.1)		
AG vs. AA			2.20 (1.23–3.91)	0.008
GG vs. AA			2.83 (1.58–5.05)	5 × 10^-4^
Multiplicative model			1.50 (1.19–188)	5 × 10^-4^
Success rate (%)	92.9	93.9		
				
**JO31**				
TT	18 (7.4)	131 (15.2)		
GT	116 (47.7)	414 (48.1)		
GG	109 (44.9)	316 (36.7)		
GT vs. TT			2.04 (1.20–3.48)	0.009
GG vs. TT			2.51 (1.47–4.30)	8 × 10^-4^
Multiplicative model			1.44 (1.15–1.79)	0.001
Success rate (%)	96.0	99.5		

Allelic discrimination was used to genotype MH30 (rs231806), CT60 (rs30807243) and JO31 (rs11571302) in 253 cases and 865 controls from northern Sweden.

### CD4^+ ^Cell Sorting

Peripheral blood mononuclear cells (PBMCs) were isolated from heparinized whole blood by density gradient centrifugation using Vacutainer CPT tubes (BD Biosciences, NJ, USA). CD4^+ ^T cells were purified by MACS cell separation using biotin-conjugated mouse anti-human CD4^+ ^monoclonal antibody (clone: RPA-T4, BD Biosciences, NJ, USA), streptavidin microbeads and MACS MS columns (MACS, Miltenyi Biotec, Germany). Average purity was found to be 97.7% by FACS analysis (FACSCalibur, BD Biosciences, NJ, USA). Streptavidin-PE antibody (BD Biosciences, NJ, USA) was used for FACS analysis.

### RNA and cDNA preparation

RNA was prepared using RNeasy Mini Kit (Qiagen, Germany) and dissolved in 40 μl of RNase-free water followed by treatment with DNase I (Ambion, Austin, TX, USA). RNA concentrations were measured (ND-1000 spectrophotometer, Nanodrop Technologies, Delaware, USA) and cDNA was prepared from 300 ng total RNA using the Reverse Transcription Reagents (TaqMan) purchased from Applied Biosystems (Applied Biosystems, Foster City, CA, USA). Each preparation step was performed according to the manufacturer's instructions.

### Gene expression

Real-time quantitative (TaqMan) PCR was performed, employing the ribosomal protein L13 (*RPL13*) as a control transcript, to investigate expression of sCTLA-4, flCTLA-4 and ICOS. Primers and probes used to measure the RNA expression were:

flCTLA-4: forward primer 5'-GAACCCAGATTTATGTAATTGATCCA-3', reverse primer 5'-CCGAACTAACTGCTGCAAGGA-3', probe 5'-(FAM)-CGTGCCCAGATTCTGACTTCCTCCTCT-(TAMRA)-3'. sCTLA-4: forward primer 5'-CATCTGCAAGGTGGAGCTCAT-3', reverse primer 5'-GGCTTCTTTTCTTTAGCAATTACATAAATC-3', probe 5'-(FAM)-ACCGCCATACTACCTGGGCATAGGCA-(TAMRA)-3'. ICOS: forward primer 5'-TGTTTCT GGCAAACATGAAGTCA-3', reverse primer 5'-GCAGAACCATTGATTTCTCCTGTTA-3', probe 5'-(VIC)-CCTCTGGTATTTCTTTCTCTTCTGCTTGCGC-(TAMRA)-3'. RPL13: forward primer 5'-CCGCTCTGGACCGTCTCAA-3', reverse primer 5'-CCTGGTACTTCCAGCCAACCT-3', probe 5'-(VIC)-TGACGGCATCCCACCGCCCT-(TAMRA)-3'. Primers were designed using Primer Express (Applied Biosystems, Foster City, CA, USA) and purchased from Applied Biosystems.

Relative expression of the transcripts was measured in the TaqMan 7900 HT Fast Real-Time PCR system (Applied Biosystems, Foster City, CA, USA) and determined by relative RNA quantification using the comparative Ct method as described by Livak and Schmittgen [10]. Briefly, the amount of target transcript normalized to an endogenous control gene and relative to a calibrator sample (total RNA prepared from PBMCs) is given by the formula: 2^-ΔΔCt^, where ΔΔCt = (Ct_target_-Ct_endogenous control_) sample in study – (Ct_target_-Ct_endogenous control_) calibrator. Expression levels of each gene were compared on the basis of the individuals CT60 genotype.

To determine the sCTLA-4/flCTLA-4 mRNA expression, we used the formula:

2^-(Ct(sCTLA-4)-Ct(flCTLA-4))^, as per [8].

### Detection of sCTLA-4 serum levels

Sandwich immunoassay for sCTLA-4 was developed in our laboratory and performed using Gyrolab Bioaffy platform (Gyros AB, Uppsala, Sweden). Briefly, the system consists of a working station performing all transfers of liquid from a microtiter plate into a CD microlaboratory, Gyrolab Bioaffy, and an integrated fluorescence detector for laser-induced fluorescence (LIF). The CD microlaboratory contains 104 identical microstructures, connected in groups of 8, to generate 104 data points. The microstructures contain a 15 nl streptavidin column to which selected capture biotinylated antibodies can be attached followed by addition of samples and detection antibodies previously labeled with Alexa Fluor 647 (Molecular Probes, Eugene, OR, USA) to form a sandwich immunoassay. Liquid introduction is facilitated by capillary force and further movement of liquids within the CD microlaboratory is driven by centrifugal force when spinning the CD. The detected fluorescence is compared with a standard curve processed in an identical fashion.

Patient serum samples stored at -20°C were slowly thawed on ice. The samples were then vortexed and centrifuged for 15 minutes at 4000 rpm and 8°C. Each sample was then diluted 1:10 (1 μl + 9 μl buffer) in microtiter platesin duplicate. Biotinylated anti-human CD152 (AS33-B; Antibody Solutions, Palo Alto, CA) was used as a capture antibody followed by a complementary anti-human CD152 antibody (BNI.3; BD PharMingen, San Diego, CA) previously labeled with Alexa Fluor 647 (Molecular Probes, Eugene, OR, USA) following the manufacturer instructions. These two matching anti-human CD152 antibodies were previously shown to be suitable for sandwich immunoassay [11]. After each addition of reagents or samples to the microstructure, the streptavidin columns were washed repeatedly using 0.01 M phosphate buffered saline, pH 7.2, containing 0.01% Tween-20 (PBS-T). CTLA4-Ig protein (Ancell, Bayport, MN) was used to generate an eight point standard curve (10 – 0.0015 ng/ml range) including a blank. sCTLA-4 protein was quantified in duplicate and calculated using Gyrolab Evaluator software(Gyros AB, Uppsala Sweden). The sensitivity limit for this assay was 0.062 ng/ml on average.

### Statistical analysis

Association analysis was performed by means of logistic regression. Genotype-based odds ratios (ORs) were calculated using individuals homozygous for the non-susceptible allele as the reference. Allele specific ORs were calculated under the assumption of a multiplicative risk model. The logistic regression was carried out in SPSS 14.0 and P-values were calculated using the Wald test. Estimation of linkage disequilibrium (LD) was performed in Haploview [12]. Markers were tested for deviation from Hardy-Weinberg equilibrium by χ^2 ^goodness-of-fit tests in the control group. Differences in the sCTLA-4/flCTLA-4 mRNA expression between the CT60 genotype groups (AA vs. AG and AA vs. GG) were examined by the Wilcoxon rank sum test.

## Results

We genotyped three SNPs in the *CTLA-4 *region in 253 cases (104 patients with AITD and 149 patients with T1D) and 865 ethnically matched controls. The investigated SNPs are in strong LD with each other (JO31-MH30 D'= 0.993 r^2 ^= 0.887, JO31-CT60 D'= 0.988 r^2 ^= 0.94, MH30-CT60 D'= 0.996 r^2 ^= 0.93). Distributions of alleles in the investigated SNPs did not deviate significantly from Hardy-Weinberg equilibrium.

For the three associated SNPs, the recessive mode of inheritance could be rejected since heterozygous individuals clearly have a greater risk compared to non-carriers (P < 0.05 for each SNP, Table 1). However, we did not observe any increased risk in homozygous individuals compared to heterozygous carriers (MH30 p = 0.26, CT60 p = 0.11 and JO31 p = 0.17) allowing for both dominant and multiplicative modes of inheritance in our AITD/T1D case-control material.

**Figure 1:**
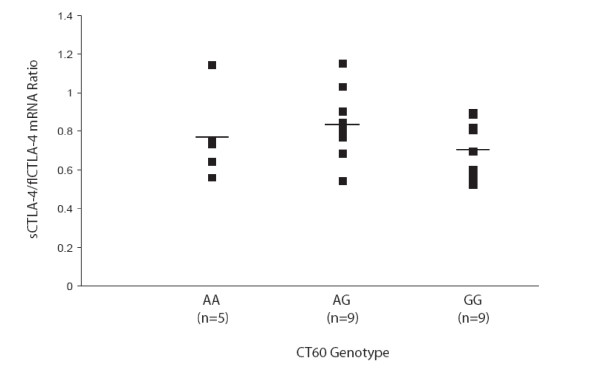
**Expression of the human sCTLA-4/flCTLA-4 mRNA ratio does not correlate with CT60 genotype**. Expression of each CTLA-4 isoform was measured by real-time quantitative PCR of RNA purified from CD4^+ ^T cells of 23 healthy volunteers. Lines represent average values within each CT60 genotype group.

**Figure 2:**
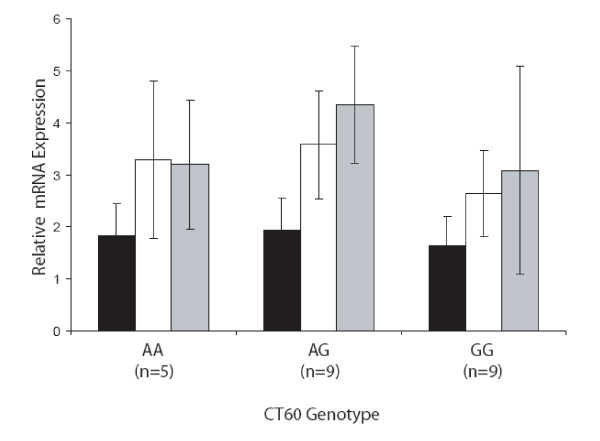
**Expression of human flCTLA-4, sCTLA-4 and ICOS mRNA does not correlate with CT60 genotype**. Expression was measured by real-time quantitative PCR of RNA purified from CD4^+ ^T cells of 23 healthy volunteers. *RPL13 *was used as an endogenous control. Error bars represent SD. Black, white and grey bars represent flCTLA-4, sCTLA-4 and ICOS mRNA expression respectively.

**Figure 3:**
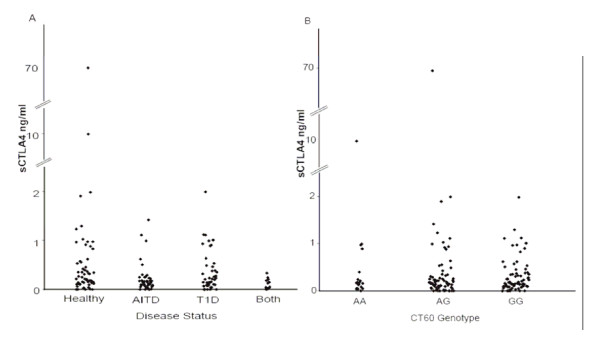
**Serum levels of sCTLA-4 protein do not correlate with disease status or CT60 genotype**. A) Levels of sCTLA-4 protein were measured with Gyrolab Workstation LIF in serum from healthy individuals (n = 59) and individuals affected with AITD (n = 43), T1D (n = 46) or both (n = 13). B) Healthy individuals were grouped according to their CT60 genotype, AA (n = 19), AG (n = 68), and GG (n = 74).

Next we analyzed the mRNA expression of flCTLA-4, sCTLA-4 and ICOS based on the CT60 genotype of 23 healthy individuals. CD4+ T cells were sorted from PBMCs using MACS cell separation. FACS analysis revealed an average purity of 97.7%. To account for the fact that the number of CTLA-4 positive cells varies between individuals, flCTLA-4 was used to normalise the sCTLA-4 values, as has been done previously [8]. No significant difference in the ratio of sCTLA-4 to flCTLA-4 mRNA expression based on the CT60 genotype could be detected (Figure 1). Both sCTLA-4 and flCTLA-4 mRNA expression, according to CT60 genotype, is given in Figure 2. ICOS expression analysis was also performed in the same individuals and no significant difference based on the CT60 genotype could be found (Figure 2). Analysis of serum sCTLA-4 protein levels in healthy individuals, AITD patients, T1D patients and individuals with both diseases revealed no significant differences when comparing disease status (Figure 3A). Subdividing the healthy individuals based on their CT60 genotype also resulted in no significant differences (Figure 3B).

## Discussion

CTLA-4 is an important negative regulator of T cell activation and we have found association between T1D/AITD and polymorphisms in close proximity to *CTLA-4 *using 253 cases (104 patients with AITD and 149 patients with T1D) and 865 ethnically matched controls from northern Sweden.

The finding that the northern Swedish frequencies of MH30*G, CT60*G and JO31*G are significantly higher in cases than controls is consistent with studies performed in other populations including the British [8,13], Italian [14], Dutch [15] and Japanese [16,17]. The high frequency of the CT60 disease susceptible G allele in our control material (61.3%) compared to the British (53.2%), Italian (48%) and Dutch (53.3%) control materials [8,14,15] might contribute to the high incidence of T1D in Sweden, which has the third highest T1D incidence in the world [18,19]. In fact, the frequency of the CT60*G allele was higher in our controls compared to the Italian Graves' disease cases (56%) and Dutch T1D cases (60%), and similar to the British Graves' disease cases (63.4%) [8,14,15]. In agreement with this, the frequency of the MH30*G allele was also higher in our controls (62.8%) compared to T1D cases in the Dutch population (60.6%) [15], and similar to Graves' disease cases in the British population (64.6%) [8]. For JO31, the frequency of the G allele in our controls (60.7%) was similar to British Graves' disease cases (61%) [8].

We have developed a sensitive sandwich immunoassay using the Gyrolab Bioaffy platform technology to detect and quantify sCTLA4 in serum samples. This technique has several advantages compared with standard ELISA assay. First, the standardization of liquid input into the detection system reduced dramatically variations within samples as well as intra- and infra-assay variation. Further, the possibility to visualize binding peaks as a histogram from the Gyros platform improved the optimisation of the assay and decreases background levels and finally the Gyros matrix technology and the particular sensitive luminescence read-out has significantly improved the detection limit of the assay compared to commercial ELISA kit (data not shown).

Analysis of sCTLA-4 and flCTLA-4 mRNA expression was performed and contrary to a previous report [8] we could not detect any significant difference in the ratio of sCTLA-4 to flCTLA-4 mRNA expression based on the CT60 genotype. Atabani *et al*. found lower sCTLA-4 to flCTLA-4 mRNA expression when comparing the CT60 GG and AA genotypes, however this analysis was performed in the smaller CD4^+ ^CD25^+ ^T cell subset [20]. No significant difference was found when analysing ICOS mRNA expression based on CT60 genotype. Our results are supported by data from Anjos *et al*. [21], who also found no effect of the CT60 polymorphism on the expression of sCTLA-4, flCTLA-4 or ICOS when looking at RNA extracted directly from PBMCs rather than isolated CD4^+ ^T cells. The studies mentioned above have used different populations of cells to measure mRNA expression of CTLA-4 isoforms. This might influence the result, since CD4^+ ^CD25^+ ^T cells constitutively express CTLA-4, while CD4^+ ^CD25^- ^T cells have a much lower expression of CTLA-4 [20]. Besides the difference in expression between CD4^+ ^CD25^+ ^and CD4^+^CD25^- ^cells, CTLA-4 is expressed by a variety of other cell types including activated CD8^+ ^T cells, activated B cells and both single and double positive thymocytes [22,23].

Analysis of serum sCTLA-4 protein levels revealed no difference in sCTLA-4 protein expression based on disease status or CT60 genotype. Taken together, these results suggest that the level of serum sCTLA-4 protein is not correlated with disease status or CT60 genotype. Measurement of sCTLA-4 protein levels in serum performed by Purohit *et al*. [24] revealed slightly elevated sCTLA-4 protein levels in T1D patients compared to controls. When grouping the individuals according to their CT60 genotype no difference in serum sCTLA-4 protein levels was found, in agreement with our findings. Oaks *et al*. [11] found higher levels of sCTLA-4 in serum from AITD patients compared to controls, however, their sample size was somewhat smaller than ours, with only 20 affected individuals and 30 healthy controls. They did not look at the effect of CT60 genotype on serum sCTLA-4 levels.

## Conclusion

In conclusion, we have shown association of three previously described polymorphisms (MH30, CT60, and JO31) in the *CTLA-4 *gene region to T1D and AITD in northern Sweden. The frequency of the CT60 disease susceptible G allele was considerably higher in our population compared to the British, Italian and Dutch populations. However, in contrast to previously published data [8], we could not find any correlation between CT60 genotype and the expression of CTLA-4 isoforms, despite using a larger sample size. Analysis of sCTLA-4 protein levels in serum revealed no correlation to either disease status or CT60 genotype. While these data do not rule out CT60 as an important polymorphism in the development of T1D and AITD, they underline the need for further studies in order to elucidate the effect of the *CTLA-4 *region on the development of T1D and AITD.

## Abbreviations

AITD, autoimmune thyroid disease; CTLA-4, cytotoxic T-lymphocyte associated antigen-4; flCTLA-4, full length cytotoxic T-lymphocyte associated antigen-4; ICOS, inducible co-stimulator; LD, linkage disequilibrium; OR, odds ratio; PBMC, peripheral blood mononuclear cell; RPL13, ribosomal protein L13; sCTLA-4, soluble cytotoxic T-lymphocyte associated antigen-4; SNP, single nucleotide polymorphism; T1D, type I diabetes mellitus.

## Competing interests

The author(s) declare that they have no competing interests.

## Authors' contributions

SM, KL, CN, KR and ME, participated in the study design, carried out the experimental work and the drafting of the manuscript. PL participated in the design of the study and performed the statistical analysis. CMC and DH conceived the study, participated in the design, and drafted the manuscript. All authors read and approved the final manuscript.

## Pre-publication history

The pre-publication history for this paper can be accessed here:


